# Change in Exercise Performance and Markers of Acute Kidney Injury Following Heat Acclimation with Permissive Dehydration

**DOI:** 10.3390/nu13030841

**Published:** 2021-03-04

**Authors:** Arpie Haroutounian, Fabiano T. Amorim, Todd A. Astorino, Nazareth Khodiguian, Katharine M. Curtiss, Aaron R. D. Matthews, Michael J. Estrada, Zachary Fennel, Zachary McKenna, Roberto Nava, Ailish C. Sheard

**Affiliations:** 1 School of Kinesiology, Nutrition, and Food Science, California State University Los Angeles, Los Angeles, CA 90032, USA; aharout4@calstatela.edu (A.H.); nkhodig@exchange.calstatela.edu (N.K.); kcurtiss2019@gmail.com (K.M.C.); amatth10@calstatela.edu (A.R.D.M.); m.estrada9208@gmail.com (M.J.E.); 2 Department of Health, Exercise, and Sports Sciences, University of New Mexico, Albuquerque, NM 87131, USA; amorim@unm.edu (F.T.A.); zfennel@unm.edu (Z.F.); zmckenna@unm.edu (Z.M.); rnavabjj@unm.edu (R.N.); 3 Department of Kinesiology, California State University San Marcos, San Marcos, CA 92096, USA; astorino@csusm.edu

**Keywords:** heat acclimation, dehydration, kidney injury, performance

## Abstract

Implementing permissive dehydration (DEH) during short-term heat acclimation (HA) may accelerate adaptations to the heat. However, HA with DEH may augment risk for acute kidney injury (AKI). This study investigated the effect of HA with permissive DEH on time-trial performance and markers of AKI. Fourteen moderately trained men (age and VO_2max_ = 25 ± 0.5 yr and 51.6 ± 1.8 mL·kg^−1^·min^−1^) were randomly assigned to DEH or euhydration (EUH). Time-trial performance and VO_2max_ were assessed in a temperate environment before and after 7 d of HA. Heat acclimation consisted of 90 min of cycling in an environmental chamber (40 °C, 35% RH). Neutrophil gelatinase-associated lipocalin (NGAL) and kidney injury molecule-1 (KIM-1) were assessed pre- and post-exercise on day 1 and day 7 of HA. Following HA, VO_2max_ did not change in either group (*p* = 0.099); however, time-trial performance significantly improved (3%, *p* < 0.01) with no difference between groups (*p* = 0.485). Compared to pre-exercise, NGAL was not significantly different following day 1 and 7 of HA (*p* = 0.113) with no difference between groups (*p* = 0.667). There was a significant increase in KIM-1 following day 1 and 7 of HA (*p* = 0.002) with no difference between groups (*p* = 0.307). Heat acclimation paired with permissive DEH does not amplify improvements in VO_2max_ or time-trial performance in a temperate environment versus EUH and does not increase markers of AKI.

## 1. Introduction

Heat acclimation allows athletes, military personnel, and occupational workers to adapt to heat stress by enhancing performance and thermal tolerance to future heat exposure [1,2] and reducing the risk of heat illness [3]. For example, heat acclimation significantly increases exercise performance when exercise is performed in a hot environment [1,2]; however, it is unclear whether heat acclimation improves exercise performance and VO_2max_ in a cool or temperate environment [4,5]. The process of heat acclimation involves various whole-body [6,7] and cellular changes [8] which are dependent on the frequency, duration, and level of thermal strain of the protocol [2,9]. It is reported that reductions in core and skin temperature typically occur by the fifth day [10,11], while increases in sweat rate usually develop by the tenth day of heat acclimation [11,12]. In addition, reduced heart rate and increased plasma volume (PV) occur within the first 3–6 days of heat acclimation [10,11,13,14]. In fact, 75–80% of these whole-body physiological changes occur within 4–7 days of heat acclimation [14,15,16]. However, the traditional 10–14-day timeframe implemented to elicit heat acclimation [7,17,18,19] may be too time consuming and impractical to employ in non-laboratory settings [13,20]. Furthermore, it has been suggested that implementing permissive dehydration during short-term heat acclimation, which is defined as restricting fluid intake during exercise to a point of modest dehydration (2–3% total body water loss), may accelerate adaptations to the heat [6].

Repeated fluid restriction during heat acclimation may expedite the onset of physiological changes because of the interrelationship between heat strain and dehydration [21,22,23]. For example, permissive dehydration during five consecutive days of heat acclimation in trained athletes increases sweat rate and electrolyte retention, expands PV, and accelerates cardiovascular and thermoregulatory adaptations similar to longer duration heat acclimation [6,20,24]. Also, the implementation of a heat acclimation and permissive dehydration protocol significantly increased lactate threshold and power output during a 20-km time-trial (TT) [20], and improved peak power output (PPO) during a VO_2max_ test in a temperate environment [13,20]. However, Neal et al. [13] concluded that heat acclimation paired with permissive dehydration did not lead to superior adaptations compared to euhydration. Consequently, the practicality and ergogenic potential of heat acclimation and permissive dehydration in accelerating adaptations or improving performance in a temperate environment is less clear and warrants further investigation.

Repeated exposure to heat stress and dehydration has been suggested to result in chronic kidney disease. Wesseling et al. [25] reported a marked prevalence of chronic kidney disease in male outdoor workers chronically exposed to dehydration and heavy physical exertion. In the context of endurance exercise, recent studies suggest an increase in early markers of acute kidney injury (AKI) when exercise is repeatedly performed in hot environments [26,27,28]. For example, markers of AKI such as plasma neutrophil gelatinase-associated lipocalin (NGAL) is significantly elevated with increasing ambient and core temperature (T_c_) and in response to dehydration in firefighters [29]. Urinary NGAL is significantly expressed in injured epithelial cells and urinary increases may indicate renal tubular damage [30]. In addition, others have shown elevated levels of kidney injury molecule-1 (KIM-1) during an ultra-distance run and following a marathon [31,32]. Kidney injury molecule-1 is a glycoprotein not expressed in healthy kidneys [33] but increases in urinary KIM-1 may indicate proximal tubule injury [34]. Despite this evidence relating to exercise, heat exposure, and increases in markers of AKI, only a few studies have explored the effect of heat acclimation/acclimatization on AKI [27,28,35,36]. In addition, it is not known if heat acclimation paired with permissive dehydration may exacerbate AKI.

Thus, the purpose of this study was to investigate the effect of moderate-term heat acclimation (7 days) with permissive dehydration on changes in exercise performance and VO_2max_ in a temperate environment in moderately trained men. A secondary purpose was to assess markers of AKI following heat acclimation and permissive dehydration. It was hypothesized that 7 days of heat acclimation paired with permissive dehydration would improve VO_2max_ and exercise performance in a temperate environment when compared to euhydration; however, this protocol would have a deleterious effect on kidney function represented by elevations in markers of AKI compared to a euhydrated state. The results of the present study will elucidate whether heat acclimation paired with permissive dehydration is efficacious in increasing endurance performance for individuals competing in a temperate environment and whether it is a safe strategy in regard to markers of kidney injury.

## 2. Materials and Methods

### 2.1. Participants

Fourteen healthy, moderately trained men were randomly assigned to either the dehydration (DEH) (*n* = 7) or euhydration (EUH) (*n* = 7) condition. All participants were engaged in moderate-to-vigorous cycling, running, and/or resistance training an average of 5 d/wk within the last year but were not competitive athletes. The participants completed a health history questionnaire to determine that they were free of cardiovascular and metabolic disease and have no current musculoskeletal injury. Written informed consent was obtained from all participants involved in the study, and procedures were approved by the California State University, Los Angeles Institutional Review Board (1066319-5).

### 2.2. Experimental Design

**Figure 1:**
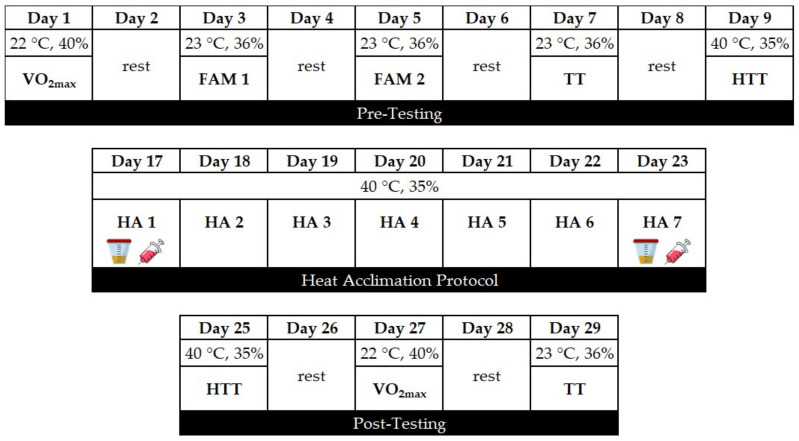
Experimental protocol undertaken by the participants. VO_2max_, maximal oxygen consumption; FAM, familiarization 16-km cycling time-trial; TT, 16-km cycling time-trial; HTT, heat tolerance test; HA, heat acclimation; urine collection and blood draw before and after the HA session.

All testing was conducted in Los Angeles, CA during January–May (average ambient temperature and humidity were equal to 16 °C and 65% RH, respectively) to ensure that subjects were not naturally heat acclimatized. Preliminary testing consisted of completion of a VO_2max_ test, two familiarization 16-km TTs, and one 16-km TT in a temperate environment (22 °C; 40% RH) followed by a heat-tolerance test in a hot and dry environment (40 °C; 30% RH). One week after preliminary testing, subjects began the 7-d heat acclimation protocol in either a EUH or DEH condition. All post-testing including assessment of VO_2max_, 16-km TT, and the heat-tolerance test, was conducted following day 7 of heat acclimation, and each trial was separated by at least 24 h. The experimental procedures are summarized in Figure 1.

All trials occurred at the same time of day, and training logs were completed by each participant prior to all visits. Participants were asked to refrain from strenuous aerobic or resistance training exercise and alcohol ingestion 24 h prior to testing. All participants were instructed to continue their regular training regimen throughout the study, and during heat acclimation they were asked to refrain from any additional lower-body exercise during the seven consecutive days. Prior to all trials, participants provided a urine sample to assess urine specific gravity (USG) (Atago 4410 Digital USG Refractometer, Atago USA Inc, Bellevue, WA, USA) which was used to determine hydration status. If participants were not well hydrated (USG ≥ 1.020 g/mL) upon arrival to the laboratory, they were asked to consume 500 mL of water, followed by a second USG assessment 30 min later. Nude body weight was assessed prior to each test after voiding using an electronic scale (Model 220, Seca, Danville, VA, USA).

### 2.3. Experimental Procedures

#### 2.3.1. Maximal Oxygen Consumption

To determine VO_2max_, participants cycled on an electronically-braked cycle ergometer (Velotron DynaFit Pro, RacerMate, Spearfish, SD) at 60 W for two minutes and work rate increased 30 W every minute until volitional fatigue. Heart rate (HR) was monitored continuously via telemetry (Polar Electro, model FS1, Woodbury, NY, USA), and rating of perceived exertion (RPE) was measured every minute using a 6–20 scale [37]. Breath-by-breath gas exchange data were continuously measured (Cosmed, Quark CPET, Rome, Italy) and VO_2max_ was recorded as the highest average value over any 15-s period. Ten minutes following the incremental test, a VO_2max_ verification test was performed at a constant load of 105% of the peak power output achieved during the incremental VO_2max_ test until the subject attained volitional fatigue [38,39]. This test was used to confirm attainment of VO_2max_ if the verification VO_2max_ was no more than 5.5% higher than the incremental value [40].

#### 2.3.2. Cycling Time-Trial

Participants completed a total of four 16-km TTs, two of which were categorized as familiarization trials, all separated by a minimum of 24 h. The TT began with a 10-min warm-up at 75 W followed by a 16 km TT (flat course; Velotron RacerMate 3-D Software, Quarq, Spearfish, SD) in a temperate environment (23 °C; 36% RH). Two familiarization trials were performed to minimize any learning effect. Participants were given verbal encouragement and were aware of the distance covered throughout the TT but were blinded to total time, power output, and HR. Heart rate and RPE were measured every 1.6 km, and water was provided ad libitum. The intraclass correlation coefficient and coefficient of variation (CV) for 16-km performance time were equal to 0.87% and 1.84%, respectively. The CV between familiarization trials is similar to values reported in previous studies [19,20,41,42].

#### 2.3.3. Heat Tolerance Test

Participants arrived at the lab after an overnight fast and ingested a standardized breakfast (unfrosted blueberry or strawberry Pop Tart) which contained 210 kcal (5 g fat, 37 g carbohydrate, and 2 g protein) immediately prior to entering the hot room. Subsequently, participants cycled for 60 min in a hot and dry environment (40 °C, 35% RH) at 35% PPO. Every 15 minutes, 250 mL of room temperature water (23 °C) was ingested to replace fluid loss with a total of 1000 mL consumed during the heat tolerance test [6,13]. Core temperature was measured using a rectal thermistor (Model 4TH, Telly Thermometer, Yellow Springs, OH, USA) which was inserted 8–10 cm beyond the anal sphincter. Core temperature, HR, thermal sensation (using a 7-point scale), and RPE were recorded every 5 min. Prior to and following the heat tolerance test, USG and dry nude body were recorded to determine hydration status and whole-body sweat rate, respectively.

#### 2.3.4. Heat Acclimation Protocol

One week after the heat tolerance test, participants initiated the 7-d heat acclimation protocol. Immediately prior to exercise, participants consumed the same standardized breakfast as described above. Participants cycled on a cycle ergometer (Monark Ergomedic 828E, Varberg, Sweden) for 90 min in a hot and dry environment (40 °C, 35% RH). A controlled hyperthermia protocol was implemented, and T_c_ was maintained within a range of 38.5–38.7 °C [20]. Participants were asked to select a workload eliciting a RPE of 15 which was maintained by adjusting either resistance or cycling cadence until a T_c_ of 38.3 °C was attained, at which point workload was adjusted by the researchers for the remainder of the 90 min protocol to ensure that the target T_c_ was maintained. Participants assigned to the EUH group consumed 1750 mL of room temperature water (23 °C) in 250 mL boluses at rest and every 15 min during each heat acclimation trial [6,13]; in contrast, individuals assigned to the permissive DEH group were not allowed to consume water during the 90 min heat acclimation bout in order to promote dehydration. Core temperature, HR, thermal sensation, and RPE were recorded every 5 min. Upon session completion, dry nude body weight and urine volume were recorded.

#### 2.3.5. Blood Sampling

Pre-and post-exercise on day 1 and 7 of heat acclimation, blood samples were drawn from the antecubital vein after 20 min of seated rest using a sterile winged push button needle and placed into sterile vacutainers containing 18 mg EDTA (Becton, Dickinson and Company, Franklin Lakes, NJ, USA). Hematocrit (Hct) was measured in triplicate by centrifuging three micro-hematocrit capillary tubes (Fisher Scientific, Pittsburg, PA) at 12,000 rpm for 5 min. Hematocrit was then determined by placing the centrifuged tubes into a micro-hematocrit capillary tube reader (Lancer, Spiracrit, St. Louis, MO, USA). The Hct was corrected by 0.96 for trapped plasma. Hemoglobin (Hb) concentration was analyzed using the HemoCue 201^+^ Analyzer (Brea, CA, USA) and measured in triplicate. Plasma volume expansion from day 1 and day 7 of heat acclimation was calculated using pre-exercise Hct and Hb measurements [43]. A 250 µL aliquot sample of plasma was analyzed in triplicate via freezing point depression using an osmometer (Advance Instruments, Inc., The Advanced Osmometer, Model, 3D3, Norwood, MA, USA).

#### 2.3.6. Assessment of Hydration Level and Markers of Kidney Function

The urine sample was collected in sterile polypropylene tubes pre-and post-exercise on day 1 and day 7 of heat acclimation. Urine KIM-1 (#ADI-900-226-0001, Enzo Life Sciences, Farmingdale, NY, USA) and NGAL (#BPD-KIT-036, BioPorto Diagnostics, Needham, MA, USA) were measured using commercially available enzyme-linked immunosorbent assays. All measurements were performed in duplicate with intra-assay CVs of 10.8% for KIM-1 and 6.8% for NGAL, respectively.

### 2.4. Statistical Analyses

All data are expressed as mean ± SD and were analyzed using IBM SPSS Version 25 (Armonk, NY, USA). Independent t-tests were used to examine differences in baseline values between groups. Normality for all variables was determined using the Shapiro-Wilks test. To identify differences in VO_2max_, TT performance, power output, HR, T_c_, sweat rate, PV expansion, RPE, and thermal sensation, a repeated measures ANOVA was used, with group (EUH vs. DEH) as the between-subjects variable and time (pre-post) as the within-subjects variable. Similarly, a repeated measures ANOVA was used to assess changes in USG, urine osmolality, NGAL, and KIM-1 from day 1 to day 7 of heat acclimation. The Greenhouse-Geisser statistic was used if the assumption of sphericity was not met. If a significant F ratio was obtained, a Tukey’s post-hoc test was used to identify differences between means. Cohen’s d was used as a measure of effect size. Statistical significance was set at *p* < 0.05. An a priori power analysis was conducted to examine the within-subjects change in TT performance from pre-to-post-heat acclimation. A sample size of twelve subjects (*n* = 6 per group) would provide sufficient power at an alpha level of 0.05 and power of 0.80.

## 3. Results

### 3.1. Heat Acclimation

There were no differences (*p* > 0.05) in any parameter between groups at baseline (Table 1). Changes in thermal strain from day 1 to day 7 of heat acclimation are shown in Table 2. Resting HR and T_c_ decreased from day 1 to day 7 of heat acclimation in both groups (*p* = 0.005 and *p* = 0.007, respectively), but there was no difference between groups (*p* = 1.00). A significant main effect for time was found for sweat rate from day 1 versus day 7 (*p* = 0.002) in both groups, but no interaction was observed (*p* = 0.293). Exercise time to attain T_c_ equal to 38.5 °C was significantly longer by day 7 in both groups (*p* = 0.012) but did not differ between groups (*p* = 0.157). Target T_c_ was well-maintained throughout heat acclimation with no T_c_ differences in either group (*p* = 0.804). There was no significant main effect for average HR from day 1 to day 7 (*p* = 0.067) and no differences were observed between groups (*p* = 0.44). The EUH group lost <1% body weight during heat acclimation from pre-post exercise on day 1 and day 7, while the DEH group lost 2.2% body weight on day 1 and 2.6% on day 7. Seven days of heat acclimation resulted in a significant PV expansion (Table 2) equal to 16% in the EUH group and 9% in DEH group (*p* < 0.05), but there was no difference between groups (*p* = 0.156). There was a significant reduction in Hct and Hb in both groups from day 1 to day 7 of heat acclimation (*p* = 0.001; *p* < 0.001), but no interaction between groups was observed (*p* = 0.277; *p* = 0.271).

### 3.2. Heat Tolerance

Only six participants in the EUH group and six participants in the DEH group performed the heat-tolerance test and the changes in physiological responses are shown in Table 3. End-exercise HR during the heat tolerance test was significantly reduced in both groups following 7 days of HA (*p* = 0.026). End-exercise HR decreased following heat acclimation in both groups (EUH 163 ± 13 b × min^−1^ to 152 ± 14 b × min^−1^; DEH 166 ± 9 b × min^−1^ to 143 ± 9 b × min^−1^). Tukey’s post-hoc test revealed a greater reduction in end-exercise HR in the DEH group (*p* = < 0.05, d = 1.75). End-T_c_ pre-post heat tolerance test was significantly reduced in both groups (*p* = 0.007), but no interaction between groups (*p* = 0.545). Both groups revealed a nonsignificant increase (5% and 6% in DEH and EUH) in sweat rate from pre-post heat acclimation (*p* = 0.412), with no difference between groups (*p* = 0.773).

### 3.3. Changes in VO_2max_ and Time-Trial Performance in Response to Heat Acclimation

There was no difference in VO_2max_ in response to heat acclimation (*p* = 0.099) and no significant interaction between groups (*p* = 0.491). However, there was a significant time effect for PPO (*p* = 0.001), yet no differences in maximal HR (*p* = 0.068) or RPE (*p* = 0.183) in either group following heat acclimation (Table 4). Seven days of heat acclimation led to a 3% improvement in TT performance in both groups (*p* < 0.01; d = 2.45, d = 3.04), but no interaction was revealed (*p* = 0.485). Peak power output during the TT was not different following 7 days of heat acclimation (*p* = 0.374); however, mean power output was significantly increased in both groups (*p* < 0.01, d = 2.64, d = 2.79), with no interaction between groups (*p* = 0.865). End-exercise HR during the TT did not change in response to heat acclimation (*p* = 0.766, *p* = 0.147). However, average RPE during the TT was significantly different in both groups following heat acclimation (*p* = 0.004), but no interaction was revealed (*p* = 0.429; Table 4).

### 3.4. Markers of Dehydration and Kidney Function

**Figure 2:**
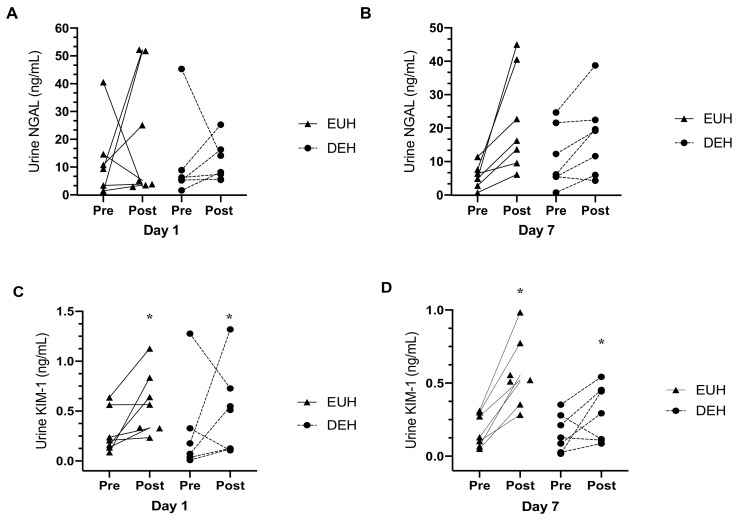
Individual data for kidney injury markers before and after 7 days of heat acclimation. (**A**) urinary neutrophil gelatinase-associated lipocalin (NGAL) response before and after day 1 of heat acclimation; (**B**) NGAL response before and after day 7 of heat acclimation; (**C**) urine kidney injury molecule-1 (KIM-1) response before and after day 1 of heat acclimation; (**D**) KIM-1 response before and after day 7 of heat acclimation; EUH, euhydrated; DEH, dehydrated; * indicating a significant main effect, *p* < 0.05. *n* = 6 for the DEH group was assessed for urine NGAL and KIM-1.

One blood sample from the EUH group was missing during data analysis. Urine specific gravity increased from pre-to-post exercise on day 1 and 7 of heat acclimation (*p* = 0.011), but there was no interaction (*p* = 0.200). Plasma osmolality significantly increased from pre-to-post exercise on day 1 and 7 (*p* = 0.009), and there was a significant interaction between groups (*p* = 0.044). Tukey’s post-hoc test revealed a greater increase in plasma osmolality in the DEH group (*p* < 0.05, d = 2.23; Table 5). Compared to pre-exercise, urine NGAL was not significantly increased following day 1 and 7 of heat acclimation (*p* = 0.113) and there was no significant interaction between groups (*p* = 0.667). However, there was a significant increase in urine KIM-1 following day 1 and 7 of heat acclimation (*p* = 0.002), but no interaction occurred (*p* = 0.307; Figure 2). It should be noted one participant’s pre-exercise urine sample from the DEH group was lost during data collection.

## 4. Discussion

Heat acclimation has repeatedly been shown to induce thermoregulatory changes which improve tolerance to exercise in a hot environment [7,24,44] However, it is unclear whether undergoing heat acclimation with permissive dehydration is an effective approach to increase endurance performance in a temperate environment more so than with adequate hydration. Further, the impact of heat acclimation with permissive dehydration on markers of AKI is unknown. Our findings suggest that (1) thermoregulatory adaptations from 7 days of heat acclimation occur independent of hydration, (2) this approach of heat acclimation does not lead to increases in VO_2max_, (3) exercise performance in a temperate environment is increased independent of hydration, and (4) a heat acclimation protocol associated with DEH did not increase markers of AKI.

There has been increased interest in examining the feasibility of short-term heat acclimation protocols [6,10,13,20,24]; however, many studies recruited well-trained participants and only two studies employed short-term heat acclimation and permissive dehydration in comparison to euhydration [6,13]. In the present study, we recruited moderately trained participants who completed a 7-day controlled hyperthermia protocol. Results showed that both groups observed a similar increase in sweat rate from day 1 to day 7 equal to 13% in the EUH group, and 8% in the DEH group. This suggests that our protocol did provide a stimulus for sudomotor adaptation, but hydration does not mediate changes in sweat rate. Resting HR significantly decreased in both groups following heat acclimation, and there was a significant reduction in resting T_c_ by the 7th day of heat acclimation, with no differences between groups. End-exercise HR and end-exercise T_c_ during the heat tolerance test were significantly reduced after heat acclimation, which corroborates previous studies [6,10,13]. Our findings demonstrate that 7 days of exercise in the heat result in cardiovascular and thermoregulatory adaptations, as shown in other studies [6,10,13]; however, permissive dehydration did not result in greater improvements in comparison to euhydration, supporting previous findings in well-trained participants [13].

As a result of exercising in the heat, participants in the DEH group lost ~2.4% of body weight on day 1 and day 7 of heat acclimation, which supports prior findings [13,20,45], and plasma osmolality significantly increased following permissive dehydration. This level of dehydration significantly increases electrolyte and water retention [46], which may lead to greater thermoregulatory and cardiovascular adaptations following heat acclimation and improved fluid balance in comparison to euhydration [6]. However, participants were dehydrated for a minimal duration during exercise and this did not induce superior changes in resting and exercise HR or T_c_, PV expansion, or improvements in exercise performance. Pethick et al., [47] reported that a 1.5–2% BW loss for 10 h during 5 days of heat acclimation did not further expand PV or improve TT performance. Additionally, Travers et al., [48] concluded that maintaining euhydration during heat acclimation reduced skin temperature and enhanced sweat rate and TT performance in the heat when compared to dehydration (~3% BW loss). Thus, dehydration during heat acclimation may not enhance heat adaptations or performance outcomes.

Permissive dehydration during short-term heat acclimation has been shown to accelerate physiological adaptations in trained participants due to increased electrolyte retention, PV expansion, and reducing cardiovascular and thermal strain [6,20]. However, our findings support data from Neal et al. [13] who showed that permissive dehydration does not further augment changes in HR, T_c,_ or sweat rate during heat acclimation or further improve VO_2max_ or exercise performance in a temperate environment. One explanation for this result is that maintaining high cardiorespiratory fitness reduces the physiological strain imposed by mild hypohydration [22] and it is apparent that trained individuals will require greater fluid regulatory strain than individuals who are less fit [49]. Our participants had a lower VO_2max_ (~51.6 mL × kg^−1^ × min^−1^) than participants in previous studies (~58.5 mL × kg^−1^ × min^−1^) [6,13], and permissive dehydration did not induce different responses compared to euhydration. However, PPO during the VO_2max_ test and mean power output during the 16-km TT were significantly higher in both the EUH and DEH group, but the mechanism leading to this improvement is unclear as VO_2max_ did not improve in either group.

Our data show that VO_2max_ did not change after 7 days of heat acclimation despite a significant PV expansion, but PPO was increased on average by 19 W, similar to Neal et al. [20] in response to heat acclimation and permissive dehydration. This result contrasts with data from Lorenzo et al. [7] who demonstrated that a 6.5% (200 mL) increase in PV resulted with a 5% increase in VO_2max_ after 10 days of exercising in the heat at 50% of VO_2max_. Our data corroborate other studies showing no effect of heat acclimation on VO_2max_ in either group even in the presence of PV expansion [13,50,51]. Karlsen et al. [50] reported a PV expansion of 15% (559 mL) following 2 weeks of heat acclimatization and a 12% expansion (503 mL) in the control group; however, there were no changes in VO_2max_ tested in a cool environment. Similarly, Keiser et al. [51] reported a PV expansion equal to 6% (201 mL) in participants who were heat acclimated with no change in the control group. Yet, these authors [51] revealed no change in VO_2max_ or TT performance in a temperate environment in either group. It is evident that any expected benefit from an expanded PV is most likely compensated by hemodilution [52] which we showed in the form of reductions in Hb and Hct that may have led to no change in VO_2max_ following heat acclimation.

Both groups exhibited similar improvements in cycling performance and mean power output in a temperate environment following heat acclimation. A previous study [7] revealed similar improvements in cycling performance in both a hot and cool environment in response to 10 days of heat acclimation. Nevertheless, in trained cyclists undergoing a 5-day heat protocol also instituting dehydration, results showed no improvement in 20-km TT performance in a temperate environment following heat acclimation [20]; however, no control group was used. Improvements in temperate TT performance following heat acclimation are believed to be due to a reduction in exercising HR and T_c_ at any given intensity throughout exercise [53,54,55]. In addition, we observed a significant increase in sweat rate during heat acclimation in both groups and this is likely to have contributed to enhanced evaporative cooling, resulting in improved blood flow to skeletal muscle and likely improved exercise performance in a temperate environment [56]. Although this was not measured in our study, another possible reason for the improvement in temperate cycling performance could be a reduction in muscle glycogen utilization during exercise following heat acclimation [57]. In response to heat stress, skeletal muscle cellular adaptations might occur which may increase mitochondrial biogenesis [58] resulting in enhanced oxidative capacity that may induce improvements in cycling performance. In summary, our data suggest that cycling performance in a temperate environment lasting approximately 25–30 min can be increased through 7 days of heat acclimation, and this adaptation is unaffected by hydration state.

Many studies [27,28,32,35,36,59] have consistently demonstrated increases in AKI markers with an acute bout of exercise completed in the heat or with dehydration. In the present study, there was a significant increase in plasma osmolality in the DEH group pre- and post-heat acclimation on day 1 and 7, but no difference in urine NGAL in either group. A significant increase in urine KIM-1 was observed in both groups from pre- to post-exercise on days 1 and 7 of heat acclimation, but no difference between groups. Although the elevations in these AKI biomarkers have been proposed to indicate intrinsic renal damage, it seems that the mild dehydration (~2.4% body weight loss) and heat strain (T_c_ = 38.5 °C) implemented in the present study were insufficient to consistently increase markers of AKI. A previous study examining firefighters [29] showed that the change in NGAL is mediated by the magnitude of increase in T_c_ and the degree of dehydration during exercise in the heat. In the present study, the elevation in T_c_ was similar between the groups, but body water loss was higher in the DEH group compared to EUH. It is plausible that this moderate dehydration (~2.4% body weight loss) did not result in significant renal ischemia and glomerular dysfunction. In addition, levels of KIM-1 increased pre-to post-exercise on day 1 and day 7 and this response occurred regardless of hydration. An increase in KIM-1 indicates proximal tubule injury and perhaps is more sensitive to AKI in the context of exercise than NGAL. Using creatinine and glomerular filtration rate as markers of AKI, Pryor et al. [28] demonstrated that 6 days of heat acclimation in healthy participants led to a reduction in the number of participants classified with AKI, but overall kidney function remained impaired. Although these two markers are frequently used in clinical settings, the observed increase may not always be indicative of kidney injury in healthy individuals exposed to mild dehydration and heat strain.

A limitation of this study is that we did not have a control group and only recruited male participants. Men and women differ in their thermoregulatory responses during exercise in the heat; therefore, the results of this study may not be generalizable to women. It is possible that because our participants were moderately fit, the 7-day heat acclimation protocol may have resulted in a training effect independent of the heat stimulus; however, we observed no changes in VO_2max_ after heat acclimation and we made sure that the participants documented and maintained their current exercise regimen throughout the study. Also, any change in VO_2max_ in our study was strengthened by using a verification test which confirmed VO_2max_ attainment before and after heat acclimation. It is possible that a learning effect may lead to increased cycling performance following heat acclimation. However, we implemented two familiarization trials prior to heat acclimation, which ensured that any learning effect would have been eliminated. The present study only examined two AKI markers; thus, the study could be strengthened by assessing markers of kidney function such as creatinine and calculated glomerular filtration rate.

## 5. Conclusions

Our results suggest that 7 days of heat acclimation does not improve VO_2max_ in moderately trained participants, yet endurance performance was significantly increased; thus, heat acclimation with permissive dehydration did not provide an additional stimulus for improving performance in a temperate environment. Moreover, heat acclimation increased KIM-1, but dehydration did not further increase markers of AKI. Maintaining hydration during exercise in the heat is important, but an increased risk of AKI may not be as prevalent for individuals who experience mild dehydration during exercise in the heat. Future research should continue to explore the incidence of kidney injury in larger groups during acute and chronic exercise in the heat with dehydration as well as examine sex differences and other kidney and inflammatory markers such as serum creatinine, glomerular filtration rate, cytokines, and cortisol.

## Figures and Tables

**Table 1:** Physical characteristics of participants (mean ± SD).

Group	Age (yr)	Body Height (cm)	Body Weight (kg)	VO_2max_ (mL × kg^−1^ × min^−1^)	VO_2max_ (L × min^−1^)
EUH	25 ± 2	174.3 ± 3.8	77.9 ± 6.7	52.9 ± 7.1	4.2 ± 0.9
DEH	26 ± 7	172.4 ± 8.8	74.1 ± 11.2	50.3 ± 8.5	3.7 ± 0.3
*p*-value	0.813	0.612	0.453	0.554	0.213

EUH, euhydrated; DEH, dehydrated.

**Table 2:** Cardiovascular and thermoregulatory changes on day 1 and day 7 of heat acclimation (mean ± SD).

Parameter	EUH		DEH	
	Day 1	Day 7	Day 1	Day 7
Resting HR (b × min^−1^)	59 ± 9	54 ± 10	58 ± 7	53 ± 6
Resting T_c_ (°C)	37.2 ± 0.2	37.0 ± 0.2	37.0 ± 0.3	36.9 ± 0.3
BW loss (%)	−0.6 ± 0.3	−0.8 ± 0.4	−2.2 ± 0.8	−2.6 ± 0.5
Sweat rate (L × h^−1^)	1.78 ± 0.18	2.01 ± 0.28	1.59 ± 0.27	1.72 ± 0.28
Average HR (b × min^−1^)	150 ± 15	148 ± 18	150 ± 7	145 ± 5
T_c_ final 60 (°C)	38.7 ± 0.2	38.6 ± 0.1	38.7 ± 0.2	38.6 ± 0.1
Time to 38.5 °C (min)	30 ± 5.1	34 ± 5.4	29 ± 9.4	40 ± 12.8
Hct (%)	46.7 ± 0.9	43.3 ± 2.7	44.6 ± 3.1	42.6 ± 2.9
Hb (g/dL)	15.8 ± 0.6	14.5 ± 0.9	14.9 ± 0.8	14.2 ± 0.8
PV expansion (%)	16.5 ± 12.0		9.2 ± 4.0	

EUH, euhydrated; DEH, dehydrated; HR, heart rate; T_c_, core temperature; BW, body weight; Hct, hematocrit; Hb, hemoglobin; PV, plasma volume.

**Table 3:** Physiological responses during the heat tolerance test following 7 days of heat acclimation (mean ± SD).

Parameter	EUH		DEH	
	Pre	Post	Pre	Post
HTT_end_ HR (b × min^−1^)	163 ± 13	152 ± 14	166 ± 9	143 ± 9 ^†^
HTT_end_ T_c_ (°C)	38.7 ± 0.4	38.4 ± 0.3	38.8 ± 0.4	38.3 ± 0.3
HTT sweat rate (L × h^−1^)	1.38 ± 0.11	1.47 ± 0.22	1.06 ± 0.25	1.11 ± 0.42

EUH, euhydrated; DEH, dehydrated; HTT, heat tolerance test; HR, heart rate; T_c_, core temperature; ^†^ indicating a significant interaction, *p* < 0.05. *n* = 6 in the EUH group and *n* = 6 in the DEH group.

**Table 4:** Effect of 7 days of heat acclimation on maximal oxygen consumption and 16-km cycling time-trial performance (mean ± SD).

Parameter	EUH		DEH	
	Pre	Post	Pre	Post
VO_2max_ (mL × kg^−1^ × min^−1^)	52.9 ± 7.1	54.8 ± 6.2	50.3 ± 8.5	51.1 ± 8.2
VO_2max_ (L × min^−1^)	4.2 ± 1.0	4.3 ± 0.9	3.7 ± 0.3	3.7 ± 0.3
HR_max_ (b × min^−1^)	186 ± 9	183 ± 6	184 ± 8	178 ± 8
VO_2max_ PPO (W)	351 ± 24	372 ± 29	332 ± 25	348 ± 36
TT time (s)	1692.9 ± 57.8	1645.4 ± 53.7	1777.3 ± 63.7	1718.3 ± 51.2
TT average PO (W)	217 ± 21	233 ± 20	191 ± 16	208 ± 15
TT end HR (b × min^−1^)	185 ± 9	183 ± 10	180 ± 13	181 ± 9
TT average RPE	15 ± 1	16 ± 1	14 ± 1	15 ± 1

EUH, euhydrated; DEH, dehydrated; VO_2max_, maximal oxygen consumption; TT, 16-km cycling time-trial; HR, heart rate; PPO, peak power output; PO, power output; RPE, rating of perceived exertion.

**Table 5:** Urine and kidney markers following 7 days of heat acclimation (mean ± SD).

**Parameter**	**EUH**		**EUH**	
	**Day 1**		**Day 7**	
	**Pre**	**Post**	**Pre**	**Post**
USG (g/mL)	1.007 ± 0.003	1.009 ± 0.004	1.005 ± 0.003	1.014 ± 0.007
Plasma osmolality (mOsm/kg)	300 ± 4	299 ± 2	298 ± 6	300 ± 3 ^†^
**Parameter**	**DEH**		**DEH**	
	**Day 1**		**Day 7**	
	**Pre**	**Post**	**Pre**	**Post**
USG (g/mL)	1.006 ± 0.005	1.012 ± 0.008	1.006 ± 0.005	1.009 ± 0.007
Plasma osmolality (mOsm/kg)	301 ± 5	307 ± 6	301 ± 4	308 ± 3 ^†^

EUH, euhydrated; DEH, dehydrated; USG, urine specific gravity; ^†^ indicating a significant interaction, *p* < 0.05. *n* = 6 for the EUH group was assessed for plasma osmolality.

## Data Availability

Data sharing is not applicable to this article. The participants in this study did not consent to access to this information by third parties for uses outside the scope of this project.
